# The Insurance Bill and the Hospitals

**Published:** 1911-11-18

**Authors:** 


### The Hospital

The Workers' Newspaper of Administrative Medicine and Institutional Life, Administration, National Insurance and Health.

No. 1323, Vol. LI. SATURDAY, NOVEMBER 18TH, 1911. PRICE ONE PENNY.

### THE INSURANCE BILL AND THE HOSPITALS.

We hope that our information may prove correct, and that the Chancellor of the Exchequer is carefully considering how best to meet the necessity for hospital benefit for the 15,000,000 persons which must arise when the National Insurance Bill becomes law. The more closely this question of hospital provision is examined the more clear does it become that the Insurance Bill as it stands, without hospital benefit, may result in additional suffering, when seriously ill, to a large number of the insured, from causes from which they were protected before such legislation came into force. The Bill, as it stands, threatens the whole of our voluntary hospitals with a financial crisis, from which they may never recover. It makes it certain that either the voluntary hospitals must be abused in the in-patient department so gravely as permanently to destroy their present financial strength, or that those insured persons, or a majority of them who need hospital care, after the passing of Mr. Lloyd George's Bill will find themselves in circumstances where they cannot obtain, without grave abuse and the acceptance of charity, the medical aid and hospital care, without which their health and even their lives may be imperilled. To state the matter thus bluntly is to show, that Parliament is face to face with a very grave position indeed in regard to this matter of hospital benefit, and that some solution must be found before the National Insurance Bill ought to become an Act of Parliament.

We are grateful to know that the whole of the managers of the voluntary hospitals throughout the United Kingdom are now alive and awake. Every day conferences of the managers of these institutions in the larger cities are being held and identical resolutions passed. These resolutions include the emphatic declaration, that the National Insurance Bill will be incomplete unless it provides for the hospital treatment which insured persons must have, if their medical needs are to be covered by Mr. Lloyd George's Bill. They properly insist that hospital treatment, as at present provided, cannot be adequately maintained if the existing sources of income from the subscriptions of workmen and employers are seriously diminished, as the most knowledgeable opinion agrees, that they are likely to be, in consequence of the provisions of the Bill as it stands. In Glasgow, in Newcastle, and in Cardiff, to mention only three large centres in Great Britain, where meetings have been called within the last few days, representative meetings of hospital authorities have been held, and everywhere identical views with those just expressed have been unanimously affirmed. Members of the House of Commons who have any regard to their plain duties and responsibilities cannot venture to neglect the representations which are being addressed to them by their most influential constituents all over the country. We welcome these signs of activity, because to be effective these meetings must be held in every centre of population and the resolutions passed and sent within the next ten days to the members of Parliament for each division represented. Then there is to be a great gathering in London of the managers and supporters of the voluntary hospitals throughout the United Kingdom. What that meeting is likely to represent and be, may be gathered from the fact, that the post on two successive days has brought intimations from ninety communities that the voluntary hospitals in those centres have unanimously decided to send their most responsible managers to London to attend the meeting, in order that the case of the voluntary hospitals, and the case of the poor insured person who really needs hospital care, may be fully considered and safeguarded, before Mr. Lloyd George's Bill passes the House of Commons. Mr. Lloyd George, we are sure, must desire to meet the case of hospital provision, and we have great hopes that a wise solution may be found. We have shown that the Bill may be strengthened, and the whole of the interests represented by the voluntary hospitals of this country, together with the needs and claims of the insured to hospital provision, may be adequately met, by a plan which will at one and the same time prove to be economical but sufficient. We publish elsewhere a full résumé of the bearings of this Bill on the voluntary hospitals, the insured, and the sick poor, which should convince everybody.

The present, so far as the hospitals and those who represent the interests of the insured are concerned, is a time for work rather than for words. We hope the great Friendly Societies may awaken in time and become fully alive to the absolute necessity for the Bill to contain provisions for hospital benefit. Unless this is done it is calculated to prove one of the most harmful and unpopular measures which have ever been conceived by the mind of man. With hospital benefits the Bill should mark an epoch, for it should prove the basis of a perfected system of health provision for all classes, under conditions which will speedily enable an adequate scheme of Poor Law reform to be passed. It should at the same time remove most, if not all, the evils which attach to our present system of medical relief and health protection.

